# Diagnostic Challenges in Atypical Presentations of Spontaneous Vertebral Artery Dissection

**DOI:** 10.7759/cureus.81372

**Published:** 2025-03-28

**Authors:** Thuy Hao Nguyen, Muhammad Ali Akhtar, Pradip Chaudhary, Bhawuk Subedi, Huda Marcus

**Affiliations:** 1 Internal Medicine, Michigan State University, Flint, USA; 2 Internal Medicine, Hurley Medical Center, Flint, USA

**Keywords:** early recognition, spontaneous vertebral artery dissection, stroke, timely treatment, vertebral artery dissection

## Abstract

Vertebral artery dissection (VAD) is a notable cause of stroke, especially in young individuals. This case reports the diagnosis of ischemic stroke in the right cerebellar hemisphere and posterior medulla on the right side, secondary to dissection of the V3 segment of the right vertebral artery of a 24-year-old female. She presented with the chief complaint of vertigo and double vision for a duration of one day. Her initial symptoms presented one day before, which were excruciating pain in her right ear and subsequent temporary hearing loss in the same ear. The patient had significant nystagmus. Computed tomography scan of the head and computed tomography angiography were negative for acute findings. Magnetic resonance imaging of the head with contrast was done, indicating an acute/subacute ischemic stroke in the right cerebellar hemisphere and posterior medulla on the right side. MRI of the neck showed occlusive dissection of the V3 segment of the right vertebral artery. Dual antiplatelet therapy was initiated with aspirin and Brilinta. This case highlights the importance of recognizing and diagnosing VAD in a timely manner, especially in patients with atypical symptoms.

## Introduction

Vertebral artery dissection (VAD) is a relatively uncommon cause of stroke in the general population, with an incidence ranging from one to five per 100,000 individuals [1]. It primarily affects those under 45 years. Dissection occurs when a weakened artery wall allows blood to accumulate, forming an intramural hematoma. This can restrict cervical artery flow directly or cause a stroke by detaching emboli that block smaller brain capillaries [2].

Several factors contribute to the formation of arterial dissection. Even minor trauma or triggers, in patients with or without risk factors, can initiate the event. Dissection has been associated with several vascular, connective tissue conditions, and several other disorders like infection, hypertension, migraine, and oral contraceptive use. Headache and neck pain were the most common initial symptoms of dissection, occurring in 57% to 90% of cases [3]. Around 70% of patients will have some neurological impairment, which may manifest later or may not present at all.

Various image tools can be used to detect VAD, such as computed tomography (CT), CT angiography (CTA), and magnetic resonance imaging (MRI). Conventional angiography, however, remains the gold standard for diagnosing VAD. VAD management depends on the extent of dissection. Reperfusion therapy, including intravenous thrombolysis, mechanical thrombectomy, or emergency stenting, is considered on a case-by-case basis.

We report a case of ischemic stroke involving the right cerebella hemisphere and posterior medulla in a 24-year-old female. The stroke was secondary to dissection of the V3 segment of the right vertebral artery. Timely recognition and diagnosis are crucial, especially in patients with atypical symptoms.

## Case presentation

A 24-year-old Caucasian female presented to the hospital with a chief complaint of vertigo and double vision of one day's duration. The night before admission, she experienced excruciating pain in her right ear, followed by hearing loss in the same ear. The next morning, the patient awoke with severe vertigo and headache radiating to her neck, associated with nausea, vomiting, double vision, impaired mobility, and swaying on the right side. Her hearing was improved.

The patient denied any speech difficulty, facial droop/asymmetry, limb weakness, bowel or bladder incontinence, any recent sick contacts, fevers, chills, or any traumatic injury to the head or neck. Past medical history included irritable bowel syndrome, and the patient was currently on Elavil and oral contraceptive pills for five years.

There was no history of trauma, vigorous activities, hypertension, diabetes, coagulopathy, malignancy, valvular heart disease, or immune disease. The patient does not smoke and rarely drinks.

On admission, the patient was alert and oriented. No focal neurological deficit or photophobia was noted. Hearing was normal to fingers rub bilaterally. The patient had significant nystagmus (horizontal, vertical, and torsional). Finger-nose and heel tests were normal, as were other neurological examinations. The patient was unable to walk due to her condition. The patient was managed with meclizine and Zofran; however, she continued to experience persistent vertigo.

CT scan of the head showed no evidence of ischemic stroke. CTA revealed the right vertebral artery hypoplastic with the impression of unremarkable findings.

**Figure 1:**
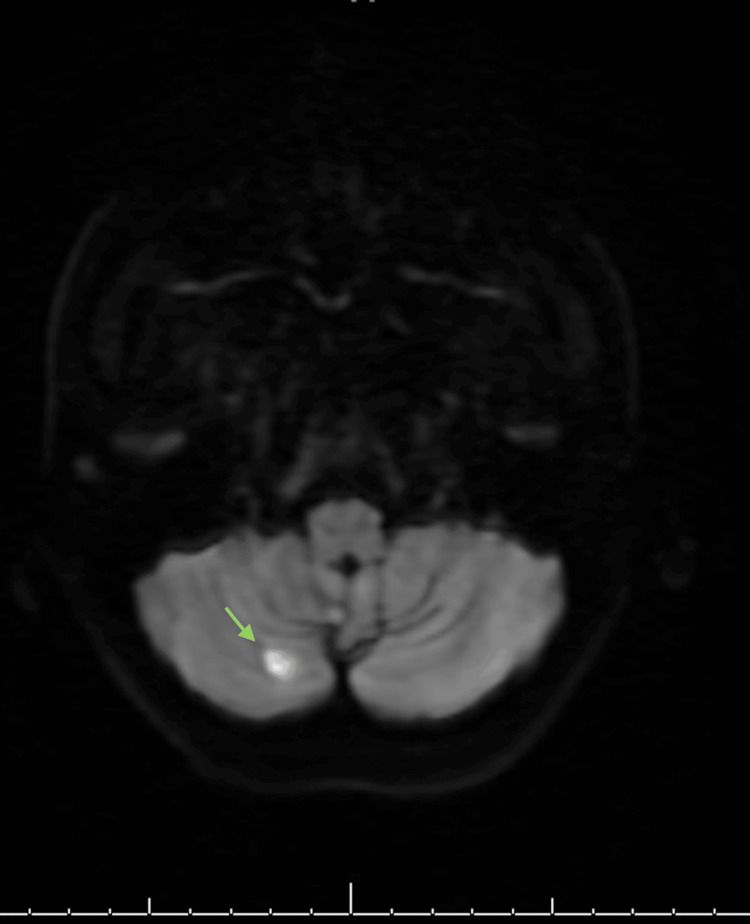
MRI of the head with contrast was done, indicating an acute/subacute ischemic stroke in the right cerebellar hemisphere.

**Figure 2:**
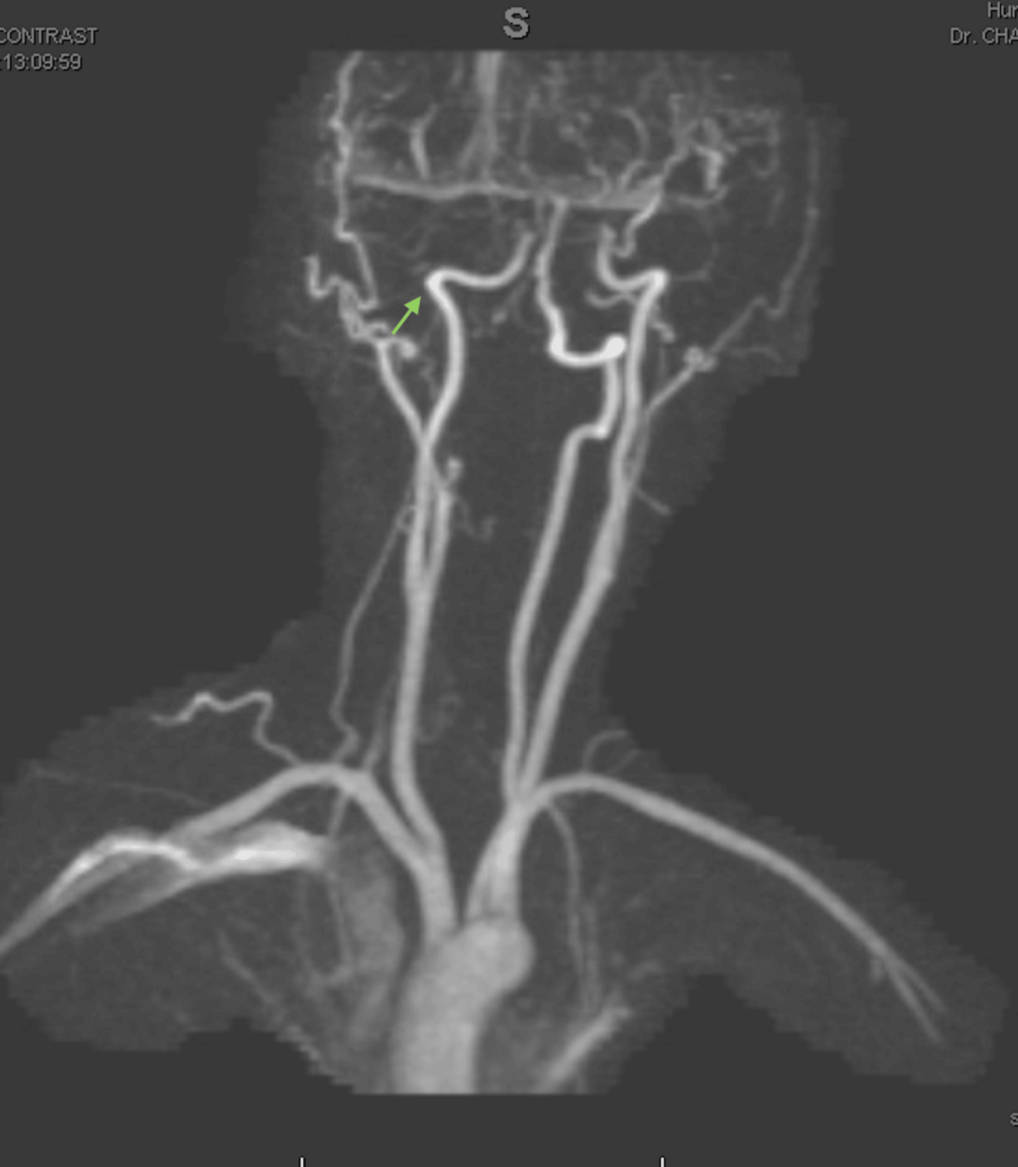
MRI of the neck showed occlusive dissection V3 segment of the right vertebral artery and reconstitution of minimal flow along the V4 segment, which may be partially attributed to retrograde flow.

Given the history of young women with no significant past medical history and negative CT and CTA results, benign positional vertigo was considered a possible diagnosis. However, the severity of her symptoms, along with persistent horizontal, vertical, and torsional nystagmus despite symptomatic treatment, raised concern. An MRI was ordered, which revealed an acute/subacute ischemic stroke in the right cerebellar and occlusive dissection V3 segment (Figures 1, 2).

Neurology was consulted, but no interventional treatment was indicated. The patient was started on a loading dose of aspirin 325 mg and Brilinta 180 mg, followed by dual antiplatelet therapy (DAPT) with aspirin 81 mg, Brilinta 90 mg twice daily, and Lipitor 80 mg. Workup for immune and blood disorders was negative. After two days of treatment, the patient’s dizziness, vertigo, and double vision resolved, and she was able to walk independently.

## Discussion

Although VAD accounts for around 2% of all ischemic strokes [4], spontaneous dissections of the carotid and vertebral arteries are a significant contributor to ischemic stroke in younger and middle-aged patients, accounting for 10% to 25% of instances. All age groups, including children, are susceptible to spontaneous dissections of the vertebral arteries, although the fifth decade of life is clearly the peak.

Various factors have been identified as possible causes of the conditions, including genetic factors such as connective tissue disorder, environmental factors such as neck hyperextension or rotation during yoga, coughing, sneezing, and chiropractic manipulation. In some cases, tobacco use, hypertension, and oral contraceptive use have been noted as potential risks of dissection; however, these factors have not been systematically studied yet [5].

Headaches account for 45% of initial symptoms of cases [6,7]. In half of the cases, the ipsilateral local symptoms linked with the cephalic pain would further help with the diagnosis. Additional symptoms may include tongue paresis, diplopia, dysgeusia, and pulsatile tinnitus. Sudden hearing loss typically affects one ear (unilateral). The incidence of bilateral hearing loss is estimated to be 0.5-1.2% in patients with vertebrobasilar ischemia and occlusive disorder, and hearing loss resulting from VAD is extremely rare [8]. Ischemic signs typically appear later, often when a stroke has already occurred.

Neurologic deficits often progress within the first few days after dissection, potentially as a complication such as ischemic stroke, which may make the diagnosis of VAD more challenging [6]. Therefore, when cervical artery dissection is suspected, early diagnostic investigation should be carried out, if possible, during the first few days following the onset of symptoms. Regardless of the type of symptoms, all patients with acute dissections of the vertebral artery have been suggested to have anticoagulation with intravenous heparin, followed by oral warfarin to prevent thromboembolic consequences, unless there are contraindications [9]. For patients with a diagnosis of transient ischemic attack (TIA) or ischemic stroke, antiplatelet therapy is more commonly prescribed [10].

In our case report, the patient initially presented with ear pain and unilateral temporary hearing loss as signs of VAD, which are atypical symptoms. Given the patient’s age, she did not fall within the typical age group vulnerable to VAD. Her only risk factor was the use of oral contraceptives, and no precipitating event was noted, suggesting that her VAD likely occurred spontaneously. Later, her primary symptoms became vertigo and nystagmus. Both her CT and CTA were negative. Her MRI later revealed acute/subacute ischemic stroke in the right cerebellar hemisphere, which was a complication of VAD. This case highlights how easily VAD can be misdiagnosed in a patient with no significant risk factors, medical history, or prominent symptoms. If ear pain and unilateral hearing loss were considered early indicators of VAD, earlier diagnosis and intervention could have been achieved. In some cases, prompt treatment might even help to prevent the occurrence of stroke.

## Conclusions

VAD is a significant contributor to stroke, particularly in younger individuals under 40 years of age. As previously mentioned, ischemia symptoms frequently manifest later, sometimes even up to one month after the dissection first happens. Thus, it is essential to keep a high level of suspicion and identify arterial dissection before the stroke occurs. This case underlines the diagnostic challenges posed by atypical symptoms such as ear pain and unilateral temporary hearing loss, particularly in patients without prominent risk factors or predisposing conditions. Early recognition of these less common presentations with minimal traditional risk factors, along with prompt imaging and treatment, is essential to preventing serious complications such as ischemic stroke. Timely diagnosis and treatment are crucial for improving patient outcomes and preventing serious complications.
